# A *Meloidogyne graminicola* C‐type lectin, Mg01965, is secreted into the host apoplast to suppress plant defence and promote parasitism

**DOI:** 10.1111/mpp.12759

**Published:** 2018-11-09

**Authors:** Kan Zhuo, Diana Naalden, Silke Nowak, Nguyen Xuan Huy, Lander Bauters, Godelieve Gheysen

**Affiliations:** ^1^ Laboratory of Plant Nematology South China Agricultural University Guangzhou 510642 China; ^2^ Guangdong Province Key Laboratory of Microbial Signals and Disease Control South China Agricultural University Guangzhou 510642 China; ^3^ Department of Biotechnology, Faculty of Bioscience Engineering Ghent University Coupure links 653 9000 Ghent Belgium; ^4^ Biology Department, College of Education Hue University 34 Le Loi Hue Vietnam

**Keywords:** apoplast, C‐type lectin, effector, *Meloidogyne graminicola*, PTI suppression, RNAi

## Abstract

C‐type lectins (CTLs), a class of multifunctional proteins, are numerous in nematodes. One CTL gene, *Mg01965*, shown to be expressed in the subventral glands, especially in the second‐stage juveniles of the root‐knot nematode *Meloidogyne graminicola*, was further analysed in this study. *In vitro* RNA interference targeting *Mg01965 *in the preparasitic juveniles significantly reduced their ability to infect host plant roots. Immunolocalizations showed that Mg01965 is secreted by *M. graminicola *into the roots during the early parasitic stages and accumulates in the apoplast. Transient expression of Mg01965 in *Nicotiana benthamiana* and targeting it to the apoplast suppressed the burst of reactive oxygen species triggered by flg22. The CTL Mg01965 suppresses plant innate immunity in the host apoplast, promoting nematode parasitism in the early infection stages.

## Introduction

Rice is an important crop, providing the staple food for a large part of the world’s population, particularly in Asia. However, rice suffers from various plant pathogens, including root‐knot nematodes (RKNs). One of the RKN species, *Meloidogyne graminicola*, commonly known as the rice RKN, is a major threat to rice agriculture, especially in Asia, causing more than 20% economic losses in rice production (Mantelin *et al*., 2017). The options for the control of *M. graminicola* are limited (Cabasan *et al*., 2012). Therefore, it is vital to understand the parasitic mechanisms of rice RKNs for the development of new and environmentally friendly disease prevention and control strategies.

Plants rely on two levels of immunity, pathogen‐associated molecular pattern (PAMP)‐triggered immunity (PTI) and effector‐triggered immunity (ETI), to resist pathogens (Jones and Dangl, 2006). When plants perceive the infection of plant pathogens, a series of immunity responses are activated, such as the mitogen‐activated protein kinase signalling cascade and calcium‐dependent protein kinase, reactive oxygen species (ROS) burst, deposition of callose, induction of defence‐related gene expression and induction of the hypersensitive response (HR) (Asai and Shirasu, 2015; Liu *et al*., 2012; Zipfel *et al*., 2004). Plant pathogens secrete diverse effectors to suppress plant immunity for their successful invasion and reproduction. Likewise, RKNs have also evolved a number of effectors that can suppress the host immune system to facilitate parasitism (Gheysen and Mitchum, 2011; Holbein *et al*., 2016). In RKNs, the effector Mi‐CRT, discovered in *Meloidogyne incognita*, was the first effector to be confirmed as capable of suppressing the host defence response, including defence‐related gene expression and callose deposition, triggered by the PAMP elf18 (Jaouannet *et al*., 2013). Since then, several effectors from different RKN species have been demonstrated to function in the suppression of host plant defence and promotion of nematode parasitism. For example, the secreted effector MiMsp40 from *M. incognita *suppresses callose deposition, defence‐related gene expression and cell death (Niu *et al*., 2016); the effector MjTTL5 of *Meloidogyne javanica* can suppress plant innate immunity by activating host ROS‐scavenging systems to eliminate ROS (Lin *et al*., 2016); the effector MeTCTP from *Meloidogyne enterolobii *suppresses plant immunity by suppressing programmed cell death in host plants (Zhuo *et al*., 2017).

Previous research on the rice–*M. graminicola* interaction has characterized the rice defence responses against RKN infection. For example, β‐aminobutyric acid‐induced resistance against *M. graminicola* in rice depends on increased basal defence, including the expression of hormone‐related genes, ROS generation and enhanced callose deposition (Ji *et al*., 2015); HR‐like reactions were detected in the incompatible, but not compatible, *M. graminicola*–rice interaction (Cabasan *et al*., 2014; Phan *et al*., 2018). More recently, evidence has emerged that *M. graminicola *secreted effectors can interfere with host immune responses. For instance, the effectors MgGPP and MgMO237 have been shown to suppress plant defences by inhibiting cell death and manipulating rice basal immunity, respectively, thereby promoting *M. graminicola *parasitism (Chen *et al*., 2018, 2017 ). Although research on *M. graminicola *effectors is limited, transcriptomic data from different life cycle stages have been reported (Chen *et al*., 2018; Haegeman *et al*., 2013; Petitot *et al*., 2016), offering a starting point to functionally characterize effectors from rice RKNs.

One candidate effector gene, *Mg01965*, derived from the *M. graminicola* transcriptome, has been shown to be expressed in the subventral oesophageal glands and is up‐regulated in the second‐stage juveniles (J2s) of *M. graminicola* (Haegeman *et al*., 2013; Petitot *et al*., 2016), suggesting its involvement in early nematode parasitism. Here, we further investigate whether Mg01965 plays a role in parasitism as an effector. We present evidence that Mg01965 is secreted into the host apoplast, in which Mg01965 functions in the suppression of plant basal immunity and promotion of *M. graminicola *parasitism.

## Results

### Sequence analysis of the *Mg01965 *gene from *M. graminicola*

**Figure 1:**
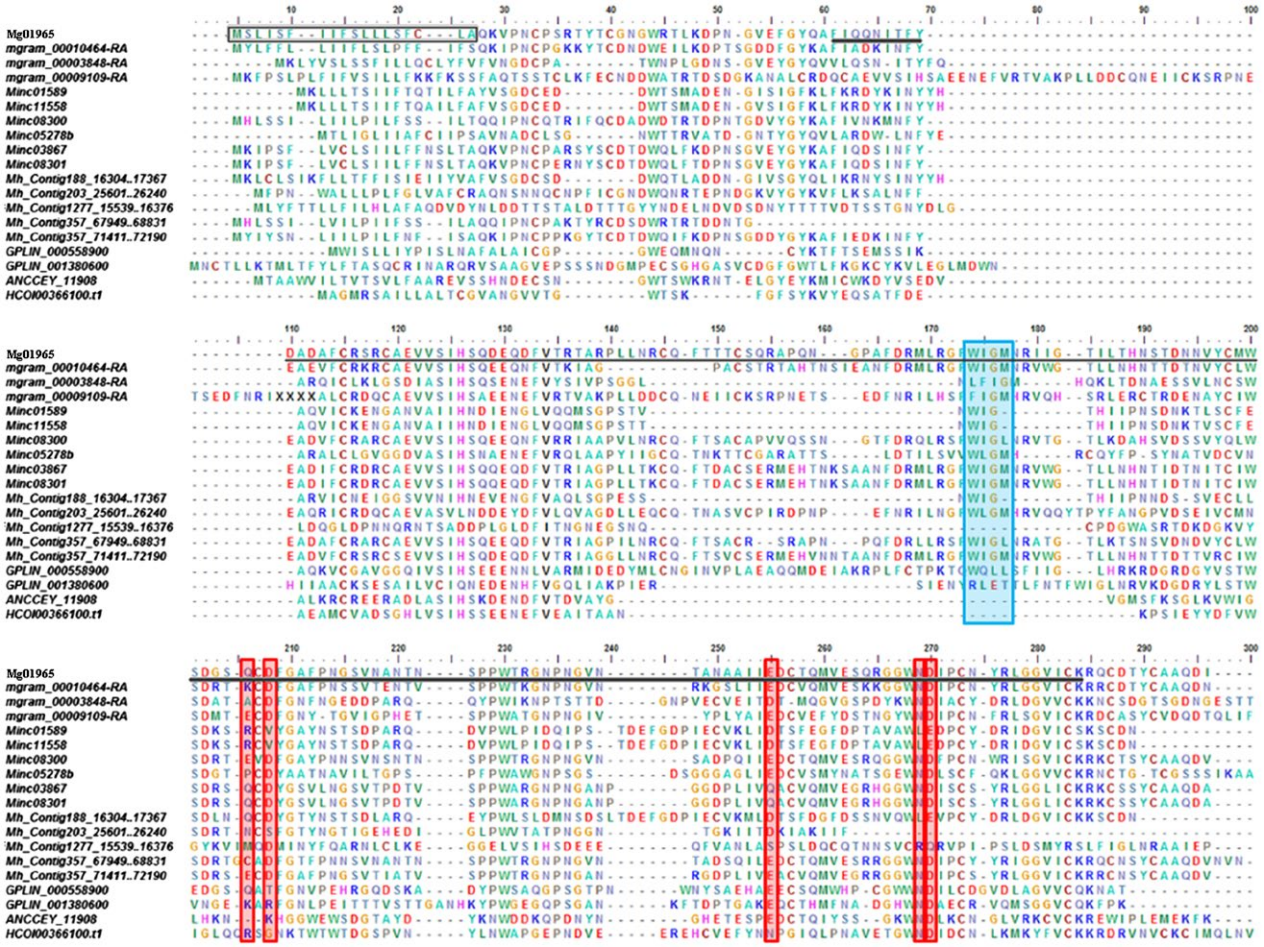
Multiple sequence alignment of the predicted Mg01965 protein with its homologues from other parasitic nematodes. Black box, predicted signal peptide; underlined, lectin domain; red boxes, key residues involved in carbohydrate binding; blue box, conserved WIGL motif; *mgram*, *Meloidogyne graminicola*; *Minc*, *M. incognita*; *Mh*, *M. hapla*; *GPLIN*, *Globodera pallida*; *ANCCEY*, *Ancylostoma ceylanicum*; *HCOI*, *Haemonchus contortus.* [Colour figure can be viewed at wileyonlinelibrary.com]

The *Mg01965* gene identified from previous work (Haegeman *et al*., 2013) includes a 681‐bp open reading frame (ORF), separated by two introns of 136 and 69 bp. The intron/exon boundaries have a conserved 5′‐GT‐AG‐3′ intron splice‐site junction (Mount, 1982). The ORF can be translated into a 226‐amino‐acid polypeptide with a predicted molecular size of 25.2 kDa. The first 18 amino acids have been predicted to function as a secretion signal and no putative transmembrane domain was found. The C‐terminal half of the protein contains a C‐type lectin (CTL)‐like domain (PF00059) from amino acid 51 to amino acid 214, in which there are five key residues that are thought to be involved in carbohydrate binding, and a characteristic CTL motif ‘WIGL’ that is important in forming hydrophobic cores in the tertiary structure of the protein (Bauters *et al*., 2017) (Figs 1 and S1, see Supporting Information). Mg01965 with the signal peptide (SP) removed (Mg01965–SP) has been predicted by PSORT II to localize in the nucleus or the cytoplasm, whereas WoLF PSORT predicted that it has an extracellular location. On the other hand, PSORT II and WoLF PSORT predicted that Mg01965 with the native SP (Mg01965+SP) localized extracellularly or in the plasma membrane, respectively (Table S1, see Supporting Information).

A BLAST search against GenBank revealed several low‐homology CTL proteins at the peptide level, for example from *Ancylostoma ceylanicum* (EPB68999, 29.9%). A BLAST search against the nematode genomes available on Wormbase Parasite (https://parasite.wormbase.org) and against several plant‐parasitic nematode (PPN) genomes resulted in 82 hits. As these CTL‐like domains are involved in a variety of functions and may be combined with several other domains, restrictions were used to look for homologues. Only sequences of approximately the same length (±50 amino acids) of Mg01965 were withheld. In addition, a predicted N‐terminal secretion signal was preferred over sequences without this signal. This left us with 18 homologous sequences, including five homologues of *Meloidogyne hapla*, three of *M. graminicola*, six of *M. incognita*, two of *Globodera pallida*, one of *Haemonchus contortus* and one of *A. ceylanicum*. A CLUSTALW alignment of the deduced amino acid sequences of Mg01965 with its homologues from other parasitic nematodes is presented in Fig. 1.

### Mg01965 affects *M. graminicola* parasitism

**Figure 2:**
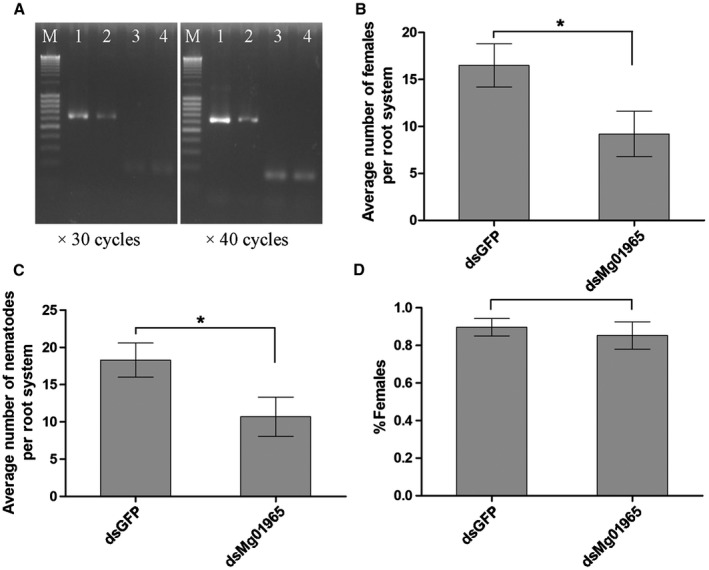
*In vitro* RNA interference (RNAi) of *Mg01965* attenuates *Meloidogyne graminicola* parasitism. (A) Semi‐quantitative reverse transcription‐polymerase chain reaction (RT‐PCR) assays analysing mRNA levels of *Mg01965* and the control gene *tubulin *in *M. graminicola* after soaking in target double‐stranded RNA (dsRNA) solution. M, size marker; 1 and 2, *Mg01965 *transcripts; 3 and 4, *tubulin *transcripts; 1 and 3, *M. graminicola *with *eGFP *dsRNA (dsGFP) treatment; 2 and 4, *M. graminicola *with *Mg01965 *dsRNA (dsMg01965) treatment. Reactions were sampled after 30 and 40 cycles. eGFP, enhanced green fluorescent protein. (B, C) RNAi with dsMg01965 showed a significant decrease in the number of females (B) and nematodes (C) compared with RNAi with dsGFP. (D) Percentage of females. Two independent experiments were performed with similar results. Data are presented as the means ± standard deviation (SD) from 10 plants. *P < 0.05, Student’s *t*‐test.

To analyse whether Mg01965 plays a role in nematode parasitism, an RNA interference (RNAi) assay was performed by soaking the nematodes in *Mg01965* double‐stranded RNA (dsRNA). Semi‐quantitative polymerase chain reaction (PCR) showed a reduction in *Mg01965 *mRNA levels after incubation with *Mg01965 *dsRNA compared with treatment with *green fluorescent protein* (*GFP*) dsRNA, but the mRNA levels of *tubulin* were not affected (Fig. 2A), showing that RNAi was successful.

Infection experiments with *Mg01965* dsRNA‐treated nematodes resulted in a significantly lower number of nematodes in rice roots compared with *GFP* dsRNA‐treated nematodes. The average numbers of females and nematodes were reduced by 44.2% and 41.5%, respectively (Fig. 2B,C). However, no significant difference in the percentage of females was found (Fig. 2D). These results indicate that Mg01965 plays a role in the initial stages of nematode infection.

### Mg01965 is secreted into the rice cell apoplast during parasitism

**Figure 3:**
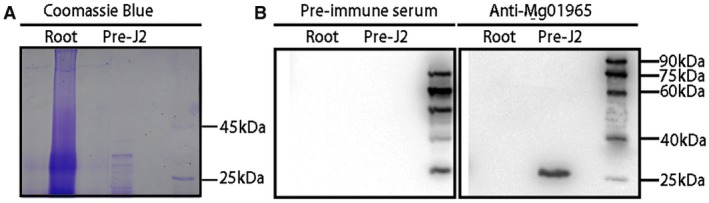
Anti‐Mg01965 serum specifically reacts with a *Meloidogyne graminicola* protein. (A) Sodium dodecylsulfate‐polyacrylamide gel electrophoresis (SDS‐PAGE) of total proteins from preparasitic second‐stage juveniles (Pre‐J2) and healthy rice roots (Root) with Coomassie blue. (B) Western blot analysis of total proteins from Pre‐J2 and Root with pre‐immune serum (left) and anti‐Mg01965 serum (right). [Colour figure can be viewed at wileyonlinelibrary.com]

To check whether Mg01965 is secreted within host plants during infection and to determine its localization in rice roots, immunolocalization was conducted on gall sections from rice plants at 3 and 5 days post‐inoculation (dpi) with *M. graminicola* using an antiserum against Mg01965. Coomassie blue staining confirmed that proteins were successfully extracted from preparasitic second‐stage juveniles (pre‐J2s) and rice roots (Fig. 3A). Western blot analysis was used to determine the serum specificity to Mg01965 using the same proteins. A clear band with the expected size of approximately 25 kDa was observed in the total protein extract from pre‐J2s, but not in that from rice roots. Meanwhile, in contrast, the control western blot treated with pre‐immune serum did not generate any visible band from the same proteins (Fig. 3B). Therefore, the antiserum against Mg01965 specifically recognizes an antigen from *M. graminicola*.

**Figure 4:**
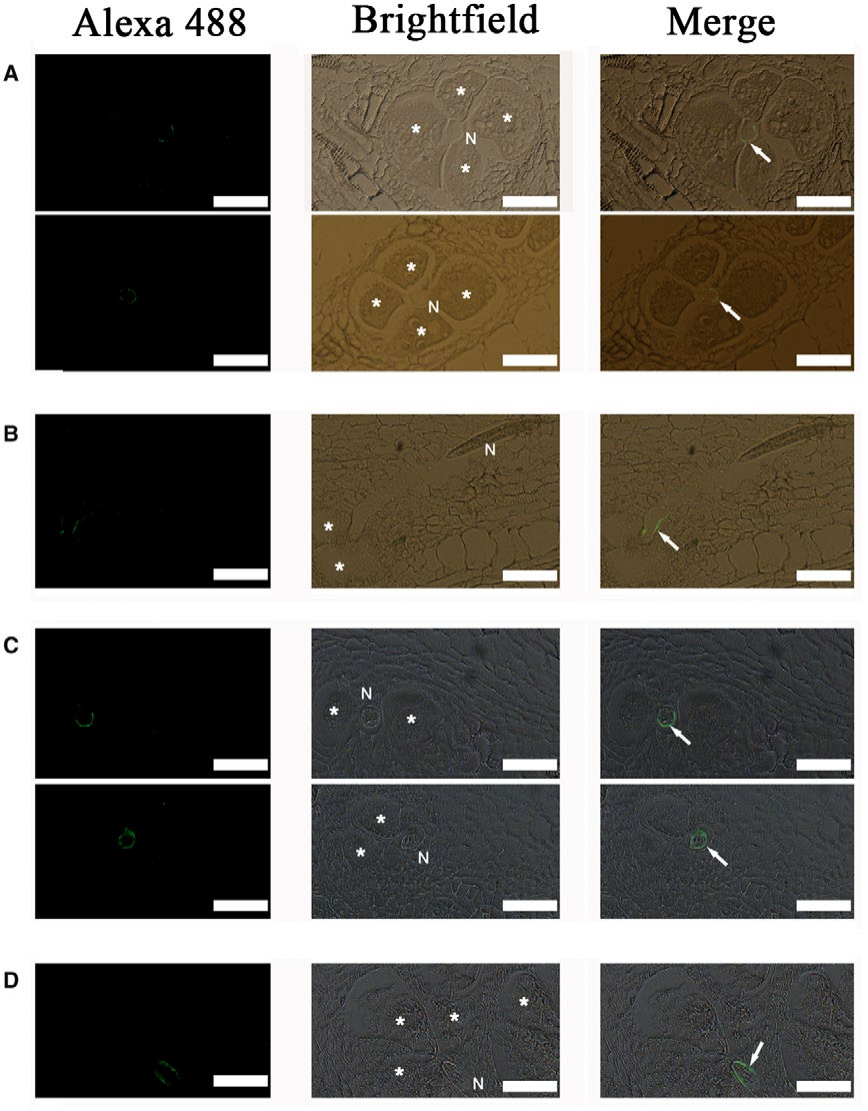
Mg01965 localization in sectioned rice root galls at 3 and 5 days post‐inoculation (dpi). (A) Localization of Mg01965 in transverse sections of rice root galls at 3 dpi, showing Mg01965 in the apoplast (white arrows). (B) Localization of Mg01965 in a longitudinal section of a rice root gall at 3 dpi, showing Mg01965 in the apoplast (white arrow). (C) Localization of Mg01965 in transverse sections of rice root galls at 5 dpi, showing Mg01965 in the apoplast (white arrows). (D) Localization of Mg01965 in a longitudinal section of a rice root gall at 5 dpi, showing Mg01965 in the apoplast (white arrow). N, nematode; asterisks, giant cells; scale bars, 50 μm. [Colour figure can be viewed at wileyonlinelibrary.com]

On both transverse and longitudinal sections, the localization of the Mg01965 protein was consistently observed in the apoplast at the *M. graminicola* interface, between the nematode and the giant cell wall, at 3 and 5 dpi with *M. graminicola* (Fig. 4). No signal was observed in the gall sections incubated with pre‐immune serum or in root sections of an uninfected plant incubated with anti‐Mg01965 serum (Fig. S2, see Supporting Information).

### Mg01965 with the SP suppresses the ROS burst

Because helminth CTLs may play a role in immune evasion (Loukas and Maizels, 2000), we investigated the possible role of the CTL Mg01965 in host defence suppression. As Mg01965 is actually secreted into the apoplast by *M. graminicola *(Fig. 4), a construct containing Mg01965+SP was used to mimic Mg01965 secretion to the apoplast. Meanwhile, a construct containing Mg01965–SP was used for comparison.

**Figure 5:**
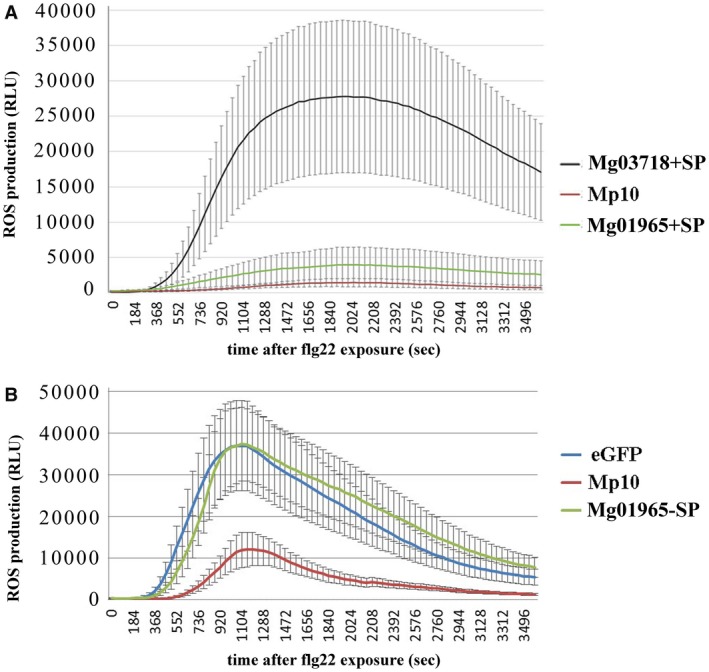
Mg01965 with signal peptide (Mg01965+SP) suppresses plant basal immunity. (A) Mg01965+SP suppresses flg22‐mediated reactive oxygen species (ROS) production in *Nicotiana benthamiana*. (B) Mg01965 without signal peptide (Mg01965–SP) does not suppress flg22‐mediated ROS production in *N. benthamiana*. Values indicated are the average relative luminescence units (RLUs) from two leaf disc replicates per leaf from eight different plants. eGFP, enhanced green fluorescent protein; Mg03718+SP, a putative effector (with similar lectin characteristics and size as Mg01965) from *M. graminicola* that cannot suppress ROS induced by flg22; Mp10, an effector of *Myzus persicae* known to suppress ROS production induced by flg22. [Colour figure can be viewed at wileyonlinelibrary.com]

Therefore, vectors expressing Mg01965+SP, Mg01965–SP, Mp10 (positive control), Mg03718+SP (negative control; another nematode protein expressed in the subventral gland and of similar size to Mg01965+SP; Haegeman *et al*., 2013) and enhanced GFP (eGFP) (negative control) were introduced into tobacco leaves through agroinfiltration. Two days after infiltration, leaf discs were collected and exposed to flg22. Similar to the positive control Mp10, *in planta* expression of Mg01965+SP reduced the flg22‐induced ROS production in comparison with the negative control Mg03718+SP. However, Mg01965–SP was unable to reduce ROS production (Fig. 5).

In addition, Mg01965+SP and Mg01965–SP were transiently expressed in *Nicotiana benthamiana* leaves together with the resistance/avirulence (*R/Avr*) gene pairs, *Cf4*/*Avr4* and *Cf9*/*Avr9*. In general, HR was observed in both Mg01965+SP and Mg01965–SP spots, indicating that there was no HR suppression (Table S2, see Supporting Information).

## Discussion

In this study, we analysed the *M. graminicola* *Mg01965* gene encoding a 164‐amino‐acid carbohydrate recognition domain with a ‘WIGL’ motif, according to CTL features (Bauters *et al*., 2017; Harcus *et al*., 2009). Lectins refer to proteins possessing at least one non‐catalytic domain that reversibly binds to a specific mono‐ or oligosaccharide, occurring in microorganisms, plants and animals (Bauters *et al*., 2017; Peumans and Van Damme, 1995). Several classes of lectins, including CTLs, galectin and hevein‐type lectins, have been demonstrated to exist in nematodes. Of these, CTLs are the most abundant lectins in nematodes (Bauters *et al*., 2017). CTLs are involved in a multitude of ‘defence’ processes, including immunity cell signalling and trafficking in vertebrates, activation of innate immunity in both vertebrates and invertebrates, and venom‐induced haemostasis (Loukas and Maizels, 2000). Several CTL proteins have been shown to be secreted by animal‐parasitic nematodes (APNs), for example, Tc‐CTL‐1 and Tc‐CTL‐4 secreted by *Toxocara canis*, and CTL1/2 released by *Heligmosomoides polygyrus* and *Nippostrongylus brasiliensis *(Harcus *et al*., 2009; Loukas *et al*., 2000, 1999). Sugar‐binding CTLs derived from APNs are thought to reduce host inflammation (Loukas and Maizels, 2000). However, the particular role of CTLs in PPNs is not well understood. In *Meloidogyne chitwoodi*, a CTL has been shown to be expressed in the subventral glands (Roze *et al*., 2008). The *Rotylenchulus reniformis* CTLs are predominantly expressed during parasitism and have been demonstrated to accumulate in the hypodermis (Ganji *et al*., 2014). In addition, exposure of *Heterodera glycines *to* CTL* dsRNA resulted in fewer parasitic nematodes in host plants (Urwin *et al*., 2002). These data suggest that CTLs of PPNs potentially play a role as effector proteins in parasitism.

In this study, bioinformatics analyses indicated that the CTL protein Mg01965 contains an N‐terminal SP, which usually aids the translocation of proteins to the endoplasmic reticulum and secretion into host plants (Elling *et al*., 2007). Moreover, previous work has found that the CTL gene *Mg01965* is expressed in the subventral glands of *M. graminicola *(Haegeman *et al*., 2013). The subventral glands produce PPN‐secreted effectors that are active during nematode penetration and in the early infection stages in roots (Davis *et al*., 2008). Therefore, we assume that Mg01965 is probably released by *M. graminicola *at the early stage of parasitism*. In planta* immunolocalization confirmed that Mg01965 was indeed secreted into the plant tissues at early parasitic stages, including 3 and 5 dpi with *M. graminicola*, and was consistently localized in the apoplast. In RKNs, several effector proteins from *M. incognita* have been shown previously to be delivered into the apoplast of infected tissues by immunocytology, including the calreticulin Mi‐CRT, the amphidial MAP‐1 protein, the aspartyl protease‐like Mi‐ASP2, CBM2‐bearing proteins, pectate lyases and the 6D4 effector (Jaubert *et al*., 2005; Vieira *et al*., 2011). In addition, three RKN effector proteins, Mi‐EFF1 from *M. incognita, *MJ‐NULG1a from *M. javanica* and MgGPP from *M. graminicola*, have been confirmed to be injected into host plants and targeted to giant cell nuclei (Chen *et al*., 2017; Jaouannet *et al*., 2012; Lin *et al*., 2013). Of these effectors, Mi‐CRT and MgGPP have been found to suppress PTI and ETI, respectively (Chen *et al*., 2017; Jaouannet *et al*., 2013). Interestingly, more recently, the *M. graminicola* effector Mg16820 has been found to be secreted in the apoplast during the migration phase of J2s, and in the cytoplasm and the nucleus of giant cells during the early development of the feeding site. When Mg16820 localizes in the apoplast, it can suppress PTI. However, Mg16820 directed to the cytoplasm is able to suppress ETI (Naalden *et al*., 2018a). All of these results suggest that RKN‐secreted effectors can suppress plant defence responses within different plant cell compartment(s), probably using different mechanisms. In this study, ROS suppression assays were performed using Mg01965+SP localizing in the apoplast and Mg01965–SP localizing in the cytoplasm to test the relation of Mg01965 and plant immunity. The results showed that Mg01965+SP can suppress the production of ROS, but Mg01965–SP cannot. In addition, neither Mg01965+SP nor Mg01965–SP can suppress HR induced by several resistance genes, suggesting that Mg01965 can suppress PTI, but not ETI. Furthermore, only Mg01965 accumulating in the apoplast can suppress PTI.

In plants, oligosaccharides are considered as priming molecules which may contribute to the immune responses against pathogens. Indeed, local increases in certain sugar levels have been observed under biotic stresses for some time (Bolouri‐Moghaddam and van den Ende, 2012; Valluru and van den Ende, 2011; Watson and Watson, 1951). Shifts in apoplastic/cell sugar content may be sensed by plants and perhaps result in the induction of immunity (Bolouri‐Moghaddam and van Den Ende, 2012; Trouvelot *et al*., 2014). Interestingly, sucrose has been proposed to be an endogenous signal to intensify immune reactions in rice (Bolouri‐Moghaddam and van den Ende, 2012). All of these data suggest that plants are able to regulate their sugar pools to serve as putative priming agents to improve immune responses (Gómez‐Ariza *et al*., 2007). In this study, the CTL Mg01965, having five out of five key residues that are thought to be involved in carbohydrate binding, was shown to be secreted into the apoplast, raising an interesting question about the role of Mg01965 in interfering with immunity by binding to certain apoplastic sugars. In addition, lectins might also bind to glycoproteins that are abundant on the outer side of the membrane (Coelho *et al*., 2017); it is therefore possible that Mg01965 suppresses immunity by binding to sugars on the plant cell membrane or in the apoplast.

Furthermore, we reduced *Mg01965* expression via *in vitro* RNAi, which led to significantly fewer nematodes in roots compared with the control groups, and no obvious difference was observed in the percentage of developing females. It is known that J2s of RKNs penetrate into host roots and migrate intercellularly before the establishment of permanent feeding sites (Wyss *et al*., 1992). Combined with the previous findings that the highest expression of *Mg01965* was in J2s, including pre‐J2s and parasitic J2s of 2 dpi (Petitot *et al*., 2016), the lower infection rate suggests that Mg01965 is an effector contributing to the early parasite–host interaction, which affects the initial infection of *M. graminicola*, but not development.

In summary, the CTL Mg01965 is released during the early infection stages of *M. graminicola* and accumulates in the apoplast, where it can suppress PTI, possibly by interference with sugar signals. Although it has been shown previously that CTLs play a role in plant parasitism (Urwin *et al*., 2002), to our knowledge, this is the first example of PPN lectins experimentally demonstrated to be secreted into the host plant as an effector. Further studies on the carbohydrate‐binding properties of the CTL Mg01965 are needed to unravel further details of its precise function.

## Experimental Procedures

### Nematode culture and plant materials

*Meloidogyne graminicola* was isolated from rice in Batangas, Philippines and cultured on rice (*Oryza* *sativa* cv. ‘Nipponbare’) in soil at 28 ºC under a 16‐h/8‐h light/dark regime in a glasshouse. Preparasitic second‐stage juveniles were collected as described previously (Naalden *et al*., 2018b).

Tobacco (*N. benthamiana*) plants were grown for 5–6 weeks at 27 ºC with 16‐h/8‐h light/dark cycles in moist universal soil (mire, garden peat and mixed nutrients). At least 1 day before infiltration, the plants were transferred to room temperature (RT) and exposed to natural light for acclimatization. After infiltration, the plants were kept at RT.

Rice plants were grown at 27 °C with 16‐h/8‐h light/dark cycles according to the protocol described in detail by Naalden *et al*. (2018b).

### Gene amplification and sequence analysis

*Meloidogyne graminicola* genomic DNA and total RNA were isolated from pre‐J2s using a Genomic DNA Purification Kit (Shenergy Biocolor, Shanghai, China) and TRIzol Reagent (Invitrogen, Carlsbad, CA, USA), respectively. First‐strand cDNAs were synthesized from 1 µg of total RNA using a BD SMART RACE cDNA Amplification Kit (Takara, Shiga, Japan), according to the manufacturer’s instructions. *Mg01965* was identified in a previous study using 454 sequencing of mRNA of pre‐J2s (contig01965; Haegeman *et al*., 2013). The full‐length coding sequences of *Mg01965* were amplified from *M. graminicola* J2 cDNA by PCR using gene‐specific primers Mg41‐F‐FL and Mg41‐R‐FL. In addition, a pair of primers, named Mg41‐DNA‐F and Mg41‐DNA‐R, was designed covering the ORF of *Mg01965* and was used to amplify the DNA sequence of *Mg01965* from *M. graminicola* DNA. All primers used in this study are listed in Table S3 (see Supporting Information).

The sequence similarity of the predicted proteins was analysed using a BLASTx search of the non‐redundant database of the National Center for Biotechnology Information (https://www.ncbi.nlm.nih.Gov/BLAST/). Meanwhile, the sequence homology was analysed in several nematode genomes and transcriptomes, including *M. incognita*, *M. hapla*, *M. graminicola*, *Bursaphelenchus xylophilus*, *G. pallida*,* A. ceylanicum* and *H. contortus*. Domains were predicted using Pfam as described by Bauters *et al*. (2017). Multiple amino acid sequences were aligned using CLUSTALW, and SP prediction used SignalP (Nielsen *et al*., 1997). The molecular mass was predicted using ProtParam (Wilkins *et al*., 1999). Transmembrane analysis was performed using TMHMM (https://www.cbs.dtu.dk/services/TMHMM/). The predicted subcellular localization of the gene was determined by PSORT II (Nakai and Horton, 1999) and WoLF PSORT (Horton *et al*., 2006).

### Plasmid construction

The full‐length coding sequence of *Mg01965* was cloned into the pGEM‐T easy vector (Promega, Tokyo, Japan) according to the manufacturer’s protocol. This construct was used as template to fuse the *Mg01965* sequence to attb sites and to ligate into the Gateway^®^ pDONR™221 vector (Thermo Fisher Scientific, San Jose, CA, USA). *Mg01965* was cloned with the start codon and with and without its native SP using the primers listed in Table S3. Ligation reactions (LR Clonase™II, Thermo Fisher Scientific) were performed to bring the *Mg01965* coding sequence to the vector pK7FWG2. As a control, a pK7WG2 derivative for the expression of eGFP was used.

### Anti‐Mg01965 polyclonal serum production and immunofluorescence localization

The anti‐Mg01965 polyclonal serum was obtained as described previously (Zhang *et al*., 2005). Briefly, the Mg01965 protein was expressed in BL21 (DE3) cells and purified using Ni^2+^NTA agarose (Merck, Darmstadt, Germany) based on the user manual. The amount and purity of the purified protein were measured using the BCA method (Tiangen Biotech, Beijing, China) and sodium dodecylsulfate‐polyacrylamide gel electrophoresis (SDS‐PAGE). The anti‐Mg01965 polyclonal serum was obtained by rabbit immunization (ABclonal, Wuhan, China).

For immunolocalization, rice galls infected with *M. graminicola* for 3 or 5 days were dissected, fixed, dehydrated and embedded in paraffin according to a previous description (Vieira *et al*., 2011). Sections were incubated in dimethylbenzene and an alcohol gradient to remove paraffin. Subsequently, the sections were immunolocalized using Mg01965 primary antibody and goat anti‐rabbit superclonal secondary antibody, Alexa Fluor 488 conjugate (Thermo Fisher Scientific), sequentially, as described previously (Chen *et al*., 2017). Finally, the sections were mounted with Fluoromount‐G (SouthernBiotech, Birmingham, UK) and observed with a Nikon ECLIPSE Ni microscope (Nikon, Tokyo, Japan).

### Western blot analysis

Rice root tissue (0.5 g) or approximately 20 000 *M. graminicola* J2s were ground in liquid nitrogen and dissolved in 1 mL of RIPA extraction buffer (2% SDS, 80 mm Tris/HCl, pH 6.8, 10% glycerol, 0.002% bromophenol blue, 5% β‐mercaptoethanol and complete protease inhibitor cocktail). After 30 min of incubation on a rotator at 4 ℃, the protein samples were centrifuged at 13500 g for 10 min to remove debris. Approximately 10 µg of total proteins were separated on a 12% SDS‐PAGE gel, and stained with Coomassie blue. The same proteins were separated on other SDS‐PAGE gels and transferred to a poly(vinylidene difluoride) (PVDF) membrane (Biorad, Shanghai, China) for western blot analysis. The membranes were blocked with 5% (w/v) nonfat dry milk for 2 h at RT, incubated with a primary mouse anti‐Mg01965 antibody at a 1 : 3000 dilution for 2 h, and then incubated with an anti‐rabbit horseradish peroxidase‐conjugated secondary antibody at a 1 : 3000 dilution for 1 h (Transgene biotech, Beijing, China). The proteins were visualized using an Immobilon Western Chemiluminescent system with Pierce ECL Western Blotting Substrate (Thermo Fisher Scientific).

### *In vitro* RNAi

*Mg01965* or *GFP* dsRNA was synthesized *in vitro* using a MEGAscript^®^ RNAi Kit (Thermo Fisher Scientific). Briefly, the sense and antisense directions of a 371‐bp *Mg01965* fragment appending the T7 sequence in the 5′ terminus were amplified using the primers Mg41‐302T7F and Mg41‐stop, or Mg41‐302F and Mg41‐T7R, in two separate PCRs. Similarly, the sense and antisense directions of a 447‐bp *GFP* fragment appending the T7 sequence in the 5′ terminus were obtained using the primers GFP‐F T7 and GFP‐R 401, or GFP‐F and GFP‐R 401 T7. Subsequently, these products were purified using a QIAquick^®^ PCR Purification Kit (Qiagen, Hilden, Germany) and used for the synthesis of dsRNA of *Mg01965* and *GFP* according to the protocol of the MEGAscript^®^ RNAi Kit. Approximately 5000 pre‐J2s of *M. graminicola* were soaked using dsRNA (1.0 mg/mL) of *Mg01965* and *GFP*, respectively, for 24 h on a rotator at 28 ℃ in the dark. To analyse the effect of RNAi, mRNAs were extracted from approximately 800 nematodes using a Nucleospin RNA plant kit (Macherey‐Nagel, Duren, Germany), according to the manufacturer’s protocol. The extracted RNA was treated with DNase I and was used as template for cDNA synthesis with oligo (dT) primer. The cDNA was used for semi‐quantitative PCR. Tubulin, a house‐keeping gene, was used to normalize the amounts of different cDNA templates. The PCR program consisted of pre‐denaturation at 94 ℃ for 5 min; 40 cycles of denaturation at 94 ℃ for 1 min, annealing at 56 ℃ for 30 s and polymerization at 72 ℃ for 30 s; with a final 5 min at 72 ℃. At 30 cycles, 20 µL of the PCR mix was withdrawn from PCR tubes and the reaction proceeded for an additional 10 cycles.

To analyse whether *Mg01965* plays a role in the parasitism of *M. graminicola*, 10 rice plants were inoculated using 200 nematodes per plant after treatment with dsRNA of *Mg01965*; another 10 rice plants were inoculated using 200 nematodes per plant after treatment with dsRNA of *GFP* as a control. At 24 h after inoculation, the plants were transferred to a hydroponic culture system with 50% of Hoagland solution (5 mm KNO_3_, 1 mm KH_2_PO_4_, 5 mm Ca(NO_3_)_2_, 2 mm MgSO_4_, 25 mm iron) to synchronize the infection process. Roots were collected at 14 dpi, stained with fuchsin acid and kept in acid glycerol. The numbers of females and juveniles were counted in each plant. Statistically significant differences between treatments were determined by performing a *t*‐test. Two independent experiments were performed.

### Defence assays

For the ROS assay after flg22 treatment, *Mg01965* with and without its native SP was cloned into the vector pK7WG2 as described above. The eGFP construct or Mg03718+SP was used as a negative control and Mp10 was used as a positive control. Mg03718+SP, another CTL, is a putative effector identified from the transcriptome of *M. graminicola *(Haegeman *et al*., 2013), with a similar size to Mg01965+SP, but unable to suppress the ROS burst induced by flg22. Mp10 is an effector of *Myzus persicae* known to suppress ROS production induced by flg22 (Bos *et al*., 2010). Leaf discs were collected and prepared for ROS assay (luminol‐based method) as described previously (Chen *et al*., 2013). Three independent experiments were performed.

For the ETI assay, two combinations of resistance/avirulence (*R/Avr*) genes, *Cf‐4/Avr4 *and *Cf‐9/Avr9 *(Thomas *et al*., 2000), were co‐infiltrated with Mg01965+SP and Mg01965–SP. Two negative controls were included in the suppression assay: *Agrobacterium tumefaciens* strain GV3101 without construct or with pK7WG2‐GFP. Agrobacteria carrying a plasmid were grown for 2–3 days in 10 mL of Luria–Bertani medium. Depending on the combination of constructs, the final concentration in the infiltration cell mixtures was adjusted to an optical density at 600 nm (OD_600_) of 0.5 for *Mg01965* and 0.5 for the *R/Avr* genes. The mixtures were spot infiltrated in *N. benthamiana* leaves of 5–6‐week‐old plants as described above, with negative controls on the same leaf as the tested effector. Per plant, two leaves were infiltrated and 20 plants were used per assay. When an HR started to appear, the response was recorded for 2 or 3 days until almost all control spots resulted in an HR. HR on a spot was considered to be suppressed when less than 50% of that spot showed cell death, following the method of Gilroy *et al*. (2011). Fisher’s exact test was used to statistically analyse the results. Each assay was performed at least twice.

## Supporting information


**Fig. S1** ****Sequence analysis of *Mg01965.* (A) cDNA coding sequence of Mg01965; the predicted signal peptide is shown in brown and underlined. (B) Protein sequence of Mg01965. The predicted signal peptide is shown in brown and underlined. The lectin domain is highlighted in green. (C) DNA sequence of *Mg01965*. The two introns are presented in red italics.
**Fig. S2** ****Immunodetection of the Mg01965 protein in sectioned rice galls. (A) Galls containing a nematode at 5 days post‐inoculation (dpi) incubated with pre‐immune serum, showing no signal. (B) Uninfected rice roots incubated with anti‐Mg01965 serum, showing no signal. N, nematode; asterisks, giant cells; scale bars, 50 μm.
**Table S1** ****Predicted subcellular localization of Mg01965.
**Table S2** ****Effector‐triggered immunity (ETI) assays.
**Table S3** ****Primers used in this study.
