# Supplementary material for: A Meloidogyne graminicola C‐type lectin, Mg01965, is secreted into the host apoplast to suppress plant defence and promote parasitism
Source: Mol Plant Pathol. 2018 Nov 9;20(3):346–55. doi: 10.1111/mpp.12759 (PMC6637863; doi:10.1111/mpp.12759)
Supplement: Supplementary file 1 — Fig. S1 Sequence analysis of Mg01965. (A) cDNA coding sequence of Mg01965; the predicted signal peptide is shown in brown and underlined. (B) Protein sequence of Mg01965. The predicted signal peptide is shown in brown and underlined. The lectin domain is highlighted in green. (C) DNA sequence of Mg01965. The two introns are presented in red italics. Fig. S2 Immunodetection of the Mg01965 protein in sectioned rice galls. (A) Galls containing a nematode at 5 days post‐inoculation (dpi) incubated with pre‐immune serum, showing no signal. (B) Uninfected rice roots incubated with anti‐Mg01965 serum, showing no signal. N, nematode; asterisks, giant cells; scale bars, 50 μm. Table S1 Predicted subcellular localization of Mg01965. Table S2 Effector‐triggered immunity (ETI) assays. Table S3 Primers used in this study. [file MPP-20-346-s001.docx]

**Supplemental Data**


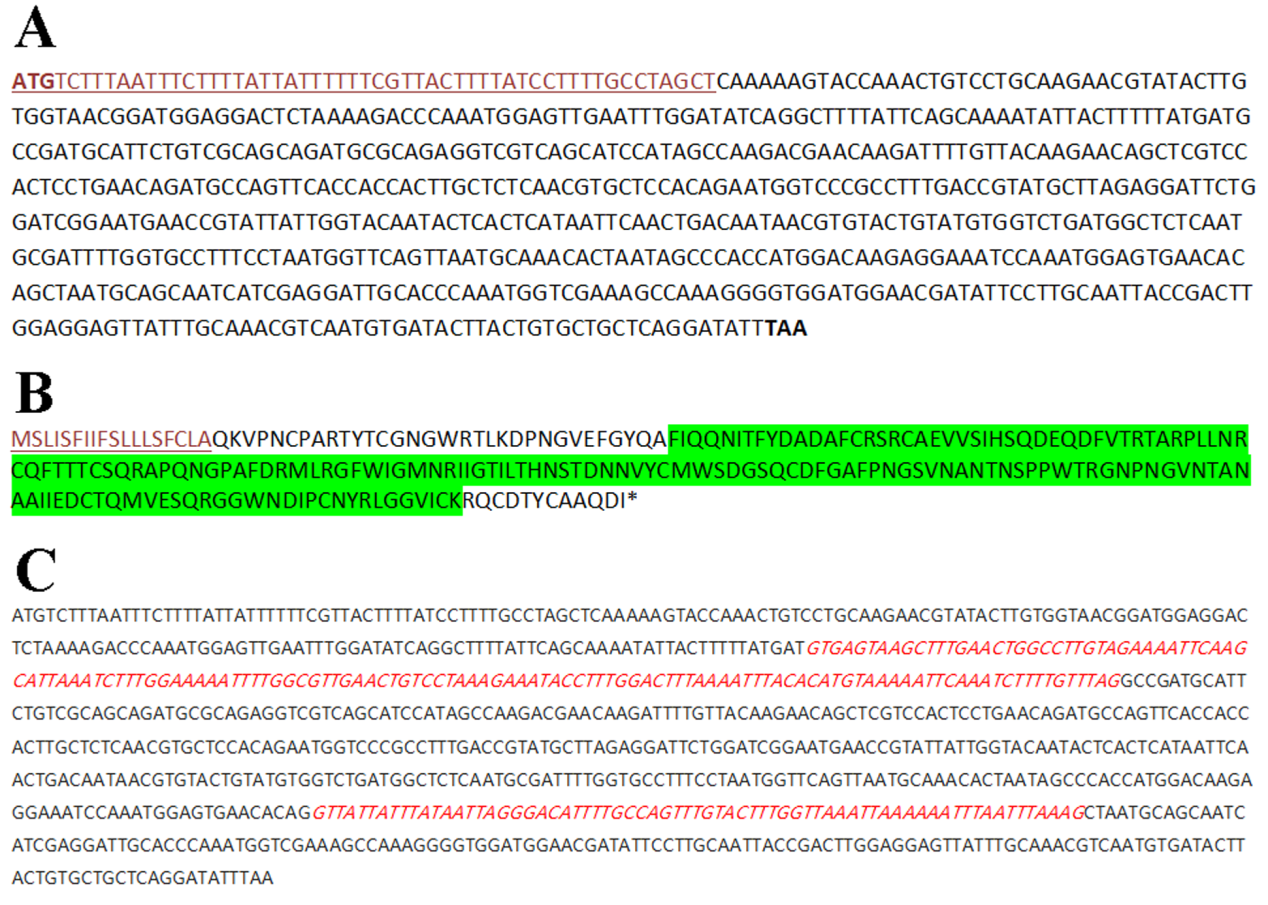


**Fig. S1 Sequence analysis of *Mg01965.*** (A) cDNA coding sequence of Mg01965, the predicted signal peptide is written in brown and underlined. (B) Protein sequence of Mg01965. The predicted signal peptide is written in brown and underlined. The lectin domain is highlighted in green. (C) DNA sequence of *Mg01965*. The two introns are presented in red italics.


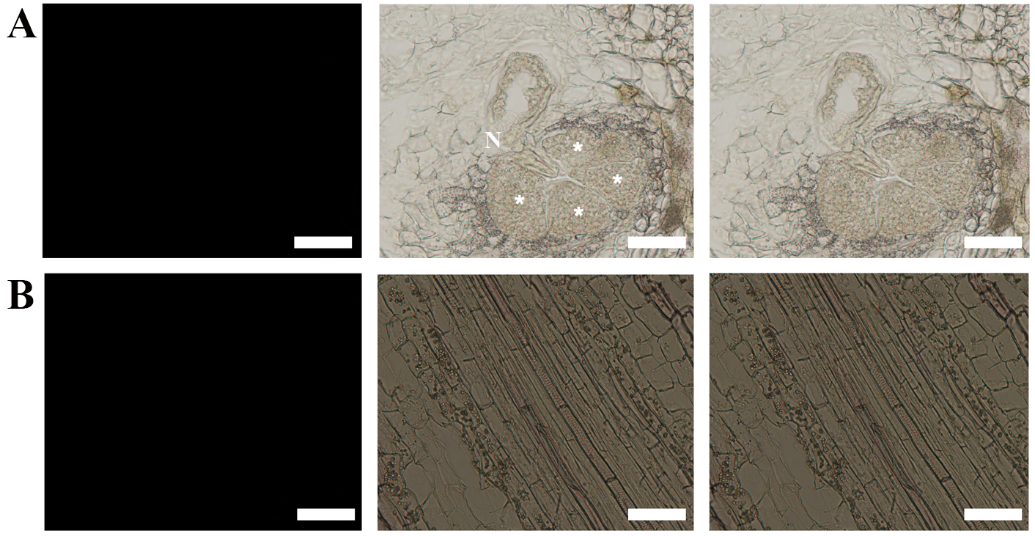


**Fig. S2** Immunodetection of the Mg01965 protein in sectioned rice galls. (A) Galls containing a nematode at 5 days post inoculation (dpi) incubated with pre-immune serum, showing no signal. (B) Uninfected rice roots incubated with anti-Mg01965 serum, showing no signal. N, nematode; asterisks, giant cells; scale bars, 50 μm.

**Supplemental Table**

**Table S1** Predicted subcellular localization of Mg01965.

| **Predicted gene** | **PSORT II prediction** | **WoLF PSORT prediction** |
| --- | --- | --- |
| Mg01965 without the signal peptide | 34.8 %: nuclear  30.4 %: cytoplasmic  26.1 %: mitochondrial  4.3 %: plasma membrane  4.3 %: peroxisomal | extracellular: 22  cytoplasmic/nuclear: 6.5  nuclear: 5.5  cytoplasmic: 4.5 |
| Mg01965 with the signal peptide | 44.4 %: extracellular, including cell wall  33.3 %: cytoplasmic  22.2 %: mitochondrial | plasma membrane: 20  extracellular: 11  lysosomal: 1 |

**Table S2** ETI assays.

| **R/Avr-gene pairs** | **Mg01965+SP** | **Mg01965-SP** | **EV** | **GFP** |
| --- | --- | --- | --- | --- |
| *Cf4/Avr4* | - (8) | - (3) | - | - |
| *Cf9/Avr9* | - (2) | - (2) | - | - |

Mg01965+SP, Mg01965 with signal peptide; Mg01965-SP, Mg01965 without signal peptide; EV, *Agrobacterium tumefaciens* strain GV3101 without construct; GFP, *A. tumefaciens* strain GV3101 with pK7WG2-GFP; -, no suppression of hypersensitive response; number in brackets, number of repetitions.

**Table S3** Primers used in this study.

| Primer | Sequence 5'- 3' | Purpose |
| --- | --- | --- |
| Mg41-DNA-F | ATGTCTTTAATTTCTTTTATTATTT | *Mg01965* genomic fragment PCR amplification |
| Mg41-DNA-R | TTAAATATCCTGAGCAGCACA |  |
| Mg41-F-FL | ATGTCTTTAATTTCTTTTAT | *Mg01965* open reading frame region PCR amplification |
| Mg41-R-FL | TTAAATATCCTGAGCAGCAC |  |
| Mg41 with SP + attB_F | aaaaagcaggcttcATGTCTTTAATTTCTTTTAT | fuse the *Mg01965* with its native signal peptide to attb sites and ligate in the Gateway^®^ pDONRTM221 vector |
| Mg41 with SP + attB_R | agaaagctgggtgAATATCCTGAGCAG |  |
| Mg41 w/o SP + attB_F | aaaaagcaggcttcATGCAAAAAGTACCAAAC | fuse the *Mg01965* without its native signal peptide to attb sites and ligate in the Gateway^®^ pDONRTM221 vector |
| Mg41 w/o SP + attB_R | agaaagctgggtgAATATCCTGAGCAG |  |
| Mg42 with SP + attB_F | aaaaagcaggcttcATGAATTTTCTCGCTAATTT | fuse the *Mg03718* with its native signal peptide to attb sites and ligate in the Gateway^®^ pDONRTM221 vector |
| Mg42 with SP + attB_R | agaaagctgggtgTGCTGAACAGTCC |  |
| Mg41-302T7F | TAATACGACTCACTATAGGGAGACTGGATCGGAATGAACCG | For the synthesis of *Mg01965* dsRNA, the sense direction of a *Mg01965* fragment appending T7 sequence in 5' terminus PCR amplification |
| Mg41-stop | TTAAATATCCTGAGCAGCAC |  |
| Mg41-302F | CTGGATCGGAATGAACCG | For the synthesis of *Mg01965* dsRNA, the antisense direction of a *Mg01965* fragment appending T7 sequence in 5' terminus PCR amplification |
| Mg41-T7R | TAATACGACTCACTATAGGGAGATTAAATATCCTGAGCAGCAC |  |
| GFP-F T7 | TAATACGACTCACTATAGGGAGAGTGAGCAAGGGCGAGGAG | For the synthesis of *eGFP* dsRNA, the sense direction of a *eGFP* fragment appending T7 sequence in 5' terminus PCR amplification |
| GFP-R 401 | CCGTCCTCCTTGAAGTCG |  |
| GFP-F | GTGAGCAAGGGCGAGGAG | For the synthesis of *eGFP* dsRNA, the antisense direction of a *eGFP* fragment appending T7 sequence in 5' terminus PCR amplification |
| GFP-R 401 T7 | TAATACGACTCACTATAGGGAGACCGTCCTCCTTGAAGTCG |  |
| Mg41nosp F | CAAAAAGTACCAAACTGTCC | Semi-quantitative PCR for *Mg01965* expression in *M. graminicola* |
| Mg41-stop | TTAAATATCCTGAGCAGCAC |  |
| Mg-Tub-F | TCTGGCATAAATAAAATAAGCGAGT | *Tubulin* expression in *M. graminicola* was used as the loading control for the semi-quantitative PCR |
| Mg-Tub-R | TCAAGATGCAACTGTTGAGGA |  |
| OsUBQ-F | CCAGTAAGTCCTCAGCCATGGAG | Rice housekeeping gene (OsUBQ) used as qRT-PCR reference |
| OsUBQ-R | GGACACAATGATTAGGGATC |  |
| OsKS4-F | TCGCATTGCGTGTGCAA | Rice defense marker gene |
| OsKS4-R | TTGGAACTTCCGACATCGAAA |  |
| OsWRKY70F | GTTTCATTTGTTTCGGAGGCC | Rice defense marker gene |
| OsWRKY70 R | TTCTCCCTATACGCCCTCTGTG |  |
| Os-WRKY13F | AGCCCATCAAGGGCTCTCCCTAC | Rice defense marker gene |
| Os-WRKY13R | TCCGTCAGCCACCGGCTCAG |  |

**REFERENCES**

**Li, R., Zhang, J., Li, J., Zhou, G., Wang, Q., Bian, W., Erb, M. and Lou, Y.** (2015) Prioritizing plant defence over growth through WRKY regulation facilitates infestation by non-target herbivores. *Elife,* **4,** e04805.

**Liu, B., Li, J.F., Ao, Y., Qu, J., Li, Z., Su, J., Zhang, Y., Liu, J., Feng, D., Qi, K., He, Y., Wang, J. and Wang, H.B.** (2012) Lysin motif-containing proteins LYP4 and LYP6 play dual roles in peptidoglycan and chitin perception in rice innate immunity. *Plant Cell,* **24,** 3406-3419.

**Shimizu, T., Nakano, T., Takamizawa, D., Desaki, Y., Ishii-Minami, N., Nishizawa, Y., Minami, E., Okada, K., Yamane, H., Kaku, H. and Shibuya, N.** (2010) Two LysM receptor molecules, CEBiP and OsCERK1, cooperatively regulate chitin elicitor signaling in rice. *Plant J.* **64,** 204-214.
